# UAV‐assisted real‐time evidence detection in outdoor crime scene investigations

**DOI:** 10.1111/1556-4029.15009

**Published:** 2022-03-09

**Authors:** Argyrios Georgiou, Peter Masters, Stephen Johnson, Luke Feetham

**Affiliations:** ^1^ Cranfield Forensic Institute, Cranfield University Defence Academy of the UK Wiltshire UK; ^2^ Cranfield University Defence Academy of the UK Wiltshire UK

**Keywords:** aerial photography, crime scene/accident investigations, forensics, real‐time evidence detection, UAV drones

## Abstract

Nowadays, a plethora of unmanned aerial vehicles (UAVs) designs that significantly vary in size, shape, operating flight altitude, and flight range have been developed to provide multidimensional capabilities across a wide range of military and civil applications. In the field of forensic and police applications, drones are becoming increasingly used instead of helicopters to assist field officers to search for vulnerable missing persons or to target criminals in crime hotspots, and also to provide high‐quality data for the documentation and reconstruction of the forensic scene or to facilitate evidence detection. This paper aims to examine the contribution of UAVs in real‐time evidence detection in outdoor crime scene investigations. It should be highlighted that the project innovates by providing a quantitative comparative analysis of UAV‐based and traditional search methods through the simulation of a crime scene investigation for evidence detection. The first experimental phase tested the usefulness of UAVs as a forensic detection tool by posing the dilemma of humans or drones. The second phase examined the ability of the drone to reproduce the obtained performance results in different terrains, while the third phase tested the accuracy in detection by subjecting the drone‐recorded videos to computer vision techniques. The experimental results indicate that drone deployment in evidence detection can provide increased accuracy and speed of detection over a range of terrain types. Additionally, it was found that real‐time object detection based on computer vision techniques could be the key enabler of drone‐based investigations if interoperability between drones and these techniques is achieved.

**Highlights**

- UAV‐assisted real‐time evidence detection in outdoor crime scene investigations.
- UAVs used as a detection tool can achieve high detections rates of nearly 100%.
- UAVs can search large areas relatively fast, thus saving man‐hours.
- UAVs can offer reliable detection capabilities over a range of terrain/vegetation types.
- Computer vision techniques can enhance drone's detection capabilities.

## 1 INTRODUCTION

### 1.1 Study's scope

The present paper aims to examine the contribution of UAV technology in evidence detection in outdoor crime scene investigations. Specifically, the study tested the efficacy of drones in real‐time object detection at a simulated outdoor crime scene, in a case where humans may fail. The efficacy in terms of accuracy and speed of detection was examined by directly comparing the drone's performance results with those obtained from a field team. It should be noted that when referring to UAV‐assisted real‐time object detection in this study, this means that the detection was performed solely by the drone operator and only by watching the drone‐based live video feed as it was displayed on the mobile device's screen. In addition, the study tested whether the performance results acquired by the drone deployment can be reproduced in different terrains and whether computer vision techniques can enhance the drone detection capabilities.

### 1.2 Literature review summary

The existing literature emphasizes the useful nature of UAVs, apart from the usability of drones in malicious acts (e.g., smuggling and spying) [1, 2, 3]. Specifically, the drone utilization today is spreading across a wide range of military [4, 5, 6, 7] and security applications [1, 2, 5, 8, 9], but also in search and rescue [10, 11] and traffic monitoring [10, 12, 13] operations. Furthermore, the contribution of UAVs is significant in sectors of mapping and land administration [14, 15], real estate [4, 8], insurance [1, 8], construction and infrastructure [8, 10], hazardous inspections and detections [8, 16, 17], agriculture [4, 8, 10, 18], telecommunications [10], media and entertainment [1, 11], e‐commerce and delivery [19, 20, 21], ecology and environmental conservation [11, 22, 23, 24, 25, 26, 27, 28], meteorology [4, 29, 30, 31], and academic research [8].

In addition, recent research [2, 32, 33, 34, 35, 36, 37, 38, 39] has underlined the usefulness of UAVs as a forensic detection tool or as a source of high‐quality data for the documentation and reconstruction of the forensic scene. The existing literature focuses mainly on the application of UAV‐based aerial photography for the detection of clandestine burials in the field of forensic archeology [33, 34, 37, 40] or for documentation purposes in crime scene or accident investigations [32, 35, 36, 38, 39]. Going one step further, Rocke et al. [41] added a Geoforensic Search Strategy (GSS) perspective to the drone deployment in the context of assessing the likelihood of detecting a buried target based on the observation of general ground conditions using technological advances in remotely sensed aerial imagery.

In addition, the recent literature puts emphasis on real‐time object and/or human detection and tracking derived from UAV‐sourced photography and videography. This is based on image processing and computer vision techniques but not under a forensic perspective (as for example in [42, 43, 44]) or highlights the usefulness of remote sensing in forensic investigations (as for example in studies [45, 46, 47, 48]).

It should be noted that the present research project innovates by quantifying the effectiveness of drones through the direct comparison with humans in the context of simulating a crime scene investigation in terms of evidence detection. Only Urbanova et al. [39] attempted to investigate drone capabilities but only by relying on drone‐recorded videos (i.e., “passive real‐time viewing”), since technical issues related to Wi‐Fi connectivity prevented them from examining the potential of drones for real‐time survey, while Sharma et al. [38] presented a qualitative comparative analysis of UAVs and traditional search methods. Both Urbanova et al. [39] and Sharma et al. [38] described the contribution of UAVs in evidence detection in crime scene investigations as beneficial.

## 2 METHODS AND MATERIALS

### 2.1 Design of experiment

The study consisted of three experimental phases: The first phase tested the usefulness of UAVs as a forensic detection tool by posing the dilemma of *humans* vs *drones*; both the drone operator by watching the live video from the drone and the field team had to detect as many items as possible in the shortest possible time. For that purpose, randomly selected objects were scattered in gradually increasing areas per scenario executed. The accuracy measured in terms of success rate of detection was determined as the primary performance criterion, while the speed measured as the time required for a full scan of the area of interest was the secondary objective. During the first phase, 16 scenarios were implemented, 4 of which focused on items that the field team had difficulty detecting.

The second phase examined the ability of a drone to reproduce the obtained performance results in different terrains; a total of 4 already implemented scenarios were conducted in two new terrains, which differed in color and morphological characteristics.

Lastly, the third phase tested the accuracy in detection by subjecting the drone‐recorded videos to computer vision techniques. For that purpose, the analysis was based on the videos acquired from the first phase, while some additional scenarios were carried out in order to investigate the software‐enhanced detection capabilities, even when the drone flew faster, and the objects were smaller. It should be highlighted that this phase does not concern real‐time detection since it was neither feasible to obtain the IP address of the drone camera nor to incorporate image processing tools into drone's software.

*Phases I and III* were implemented in May 2019 during morning or afternoon hours. The weather was mostly sunny or partly cloudy with winds up to 18 kph, and therefore, the drone operation was not affected since DJI SPARK™ can withstand wind speeds of up to 28 kph [49]. *Phase II* was conducted in June 2019 under similar weather and daylight conditions.

Each round (i.e., scenario) of the experiment was prepared by scattering the items in the area of interest. Both the field team and the drone operator were in the adjacent parking site without having direct view to the sports pitch in order to avoid having prior knowledge of the objects' position. In addition, both the field team and the drone operator were aware of the scanning patterns to be followed before entering the scene but none of them knew the number of the objects included in each scenario.

The time started to count when the field team or drone entered the scene and stopped when they left the scene. The time required to upload the CSV files containing the drone's flight plan through the Litchi website (as mentioned in Section 2.6) did not count since this process takes only 1–2 min and the preparation can be done prior to arrival on scene, as it really happened. Lastly, it should be noted that when a searcher of the field team detected an object, he simply had to raise his hand and continue the search without having to stop to collect it.

### 2.2 Study area

The experiment was conducted in the approved areas for outdoor drone flying of the Defence Academy of the United Kingdom in Shrivenham after receiving permission from the Defence Academy Site Security. All study areas were free of spatial constraints, such as trees, which may impede the drone's accessibility capabilities or limit visibility due to vegetation cover, while 4G signal and/or Wi‐Fi networks were available at all times.

In specific, the flights for *Phases I and III* were undertaken at a sports pitch, which was covered with dense, green, and short grass (approximately 5–6 cm tall). The field had a well‐groomed appearance characterized by a smooth and even cut without having a remarkable amount of grass clippings from lawn mowing or dead grass spots. The extent of the area used to implement the *Phase I* scenarios ranged between 30 m × 30 m and 30 m × 85 m.

*Phase II* was conducted in two areas of the Explosives Research Detonation Area (ERDA) with different terrain in terms of color and morphology. The first area was a relatively even surface of red clay soil with scattered short green plants (5–10 cm tall), while the second field was grass‐covered (grass height: 15–25 cm) with large quantities of grass clippings residues and dead grass or uneven surface spots. The area used for the implementation of *Phase II* scenarios was 30 m × 30 m and 30 m × 60 m.

### 2.3 Objects

The objects used in this study were 2 mm thick foam pads in order to avoid the direct detection by the field team from a distance, since the flat surface of the first phase's study area is not the common case; in real life, uneven surfaces and/or the presence of natural or artificial barriers can limit visibility.

The shape of the items was decided to be square for reasons of convenience, while the size was determined after running some trials with the field team in order to define the threshold below which humans might have difficulty in detection; in this way, it was possible to test whether the drone deployment could effectively contribute to evidence detection. Therefore, it was determined that 5 cm × 5 cm was the appropriate size for the study.

In addition, it was decided that the objects would be randomly selected from 8 predefined colors: red‐blue‐yellow (primary), green‐purple‐orange (secondary) plus black and white; each color corresponded to 10 objects out of the total 80 used in these multi‐colored scenarios of *Phase I* (i.e., 12.5%). It should be highlighted that only the color that the field team had difficulty to detect (i.e., black) was used for the last 4 single‐colored scenarios of the first phase. Lastly, the number of items used per multi‐colored scenario of *Phase I* was randomly selected, ranging between 5 and 10, while each single‐colored scenario contained 10 (black) objects.

### 2.4 Field team

The field team consisted of two military officers with accumulated experience in Counter‐IEDs activities and aircraft accident investigations.

### 2.5 UAS

#### 2.5.1 Aircraft and payloads

The unmanned aerial vehicle used in the experimental phase was a DJI SPARK™, which is a low‐cost drone that incorporates all the signature technologies of DJI. SPARK™ is equipped with vision (VPS) and global positioning systems (GPS). As for the camera, which is flush with the aircraft, SPARK™ features a 1/2.3″ CMOS sensor that delivers 12MP photographs and FHD 1080p videos, while the wide‐angle lens (with 25 mm equivalent focal length) provides sharp and vibrant color images with reduced chromatic aberration and distortion [49].

#### 2.5.2 Command and control element and communication data link

The SPARK™ remote controller was paired with the drone, and by using its advanced Wi‐Fi signal transmission system, it was possible to operate both the aircraft and the gimbal camera at a maximum distance of 2 km [49]. In addition, the controller was attached and wirelessly connected to a Samsung Galaxy S8+ mobile phone, which was used to display the live video stream.

#### 2.5.3 Launch and recovery element

The equipment needed to takeoff and land the DJI SPARK™ was a circular launch pad, as the drone can ascend and descend vertically.

#### 2.5.4 Human element

The drone operator was a Research Fellow in Imaging and Autonomous Systems Centre of Electronic Warfare, Information and Cyber of Cranfield University. In the past, the drone operator has performed relevant tasks in similar research projects.

### 2.6 Software used

#### 2.6.1 Microsoft (MS) Excel

Waypoints were automatically generated in MS Excel for the full scan of the area of interest. Flight paths were generated by a custom, in‐house developed, MS Excel spreadsheet using VBA macros for spherical geometry calculations.

Moreover, MS Excel was used to randomly select the number and the color of the objects used in each scenario, as well as their position in the study area.

#### 2.6.2 Litchi

The Litchi website was used to upload the flight plan (in CSV format files as created by MS Excel) and to save the flight plan in order to be available on the phone application via the cloud, while the Litchi android application is used in order to fly autonomously the DJI drones such as the SPARK™.

#### 2.6.3 DJI GO 4 android application

DJI drones cannot takeoff in Authorization Zones (e.g., military zones), such as the Defence Academy of the UK, and users are required to unlock the flight restriction through their DJI‐verified account [50]. Hence, the DJI GO 4 application was used to get permission to operate in the no‐fly zone of the study area.

#### 2.6.4 MATLAB (version R2019a)

A color detection algorithm was created to recognize colors in the drone‐sourced videos and thus to facilitate object detection. MATLAB code was run in the pre‐recorded videos, as neither the IP address of the drone camera could be obtained nor the drone itself could execute the script in order to achieve the real‐time implementation of the algorithm. The in‐house developed algorithm was able to identify red‐green‐blue‐white, the orange was recognized in case of simultaneous detection of red and yellow, while purple and black can be detected by increasing the sensitivity for blue color identification.

### 2.7 Implemented scenarios

During the first part of *Phase I*, random selected objects (as referred to in Section 2.3) were scattered over gradually increasing areas of the sports pitch (as mentioned in Section 2.2). The study area increased from 30 m × 30 m to 30 m × 85 m, by following a 5 m increase along its variable dimension per repetition of the experiment; hence, 12 scenarios corresponding to these areas were prepared. The second part of *Phase I* focused on objects that the field team had difficulty detecting (as mentioned in Section 2.3). For this purpose, 10 black objects were randomly dispersed in areas of 30 m × 60 m and 30 m × 85 m; 2 scenarios were created for each different area. The scenarios performed in *Phase II* were the same as those of *Phase I* (Part 1) corresponding to 30 m × 30 m and 30 m × 60 m areas.

### 2.8 Search patterns

**FIGURE 1:**
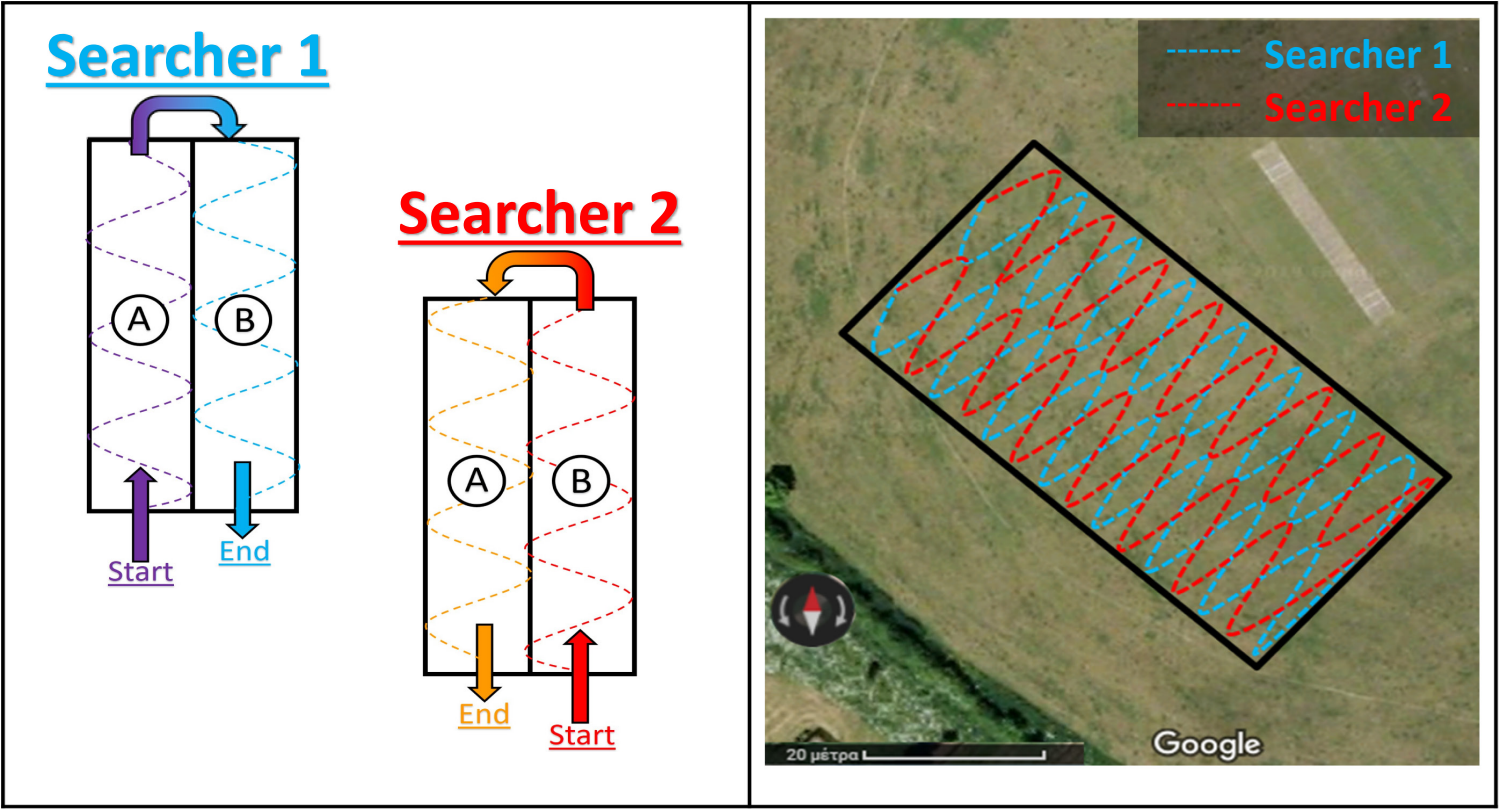
Field team's search pattern in a 30 m × 60 m area [51]

The field team decided to divide the area in half (Zone A and B). Then, the searchers dealt with each zone individually by following a strip search pattern; after conducting a detailed search of their zones, they switched halves in order to ensure that the area would be double‐checked (Figure 1).

**FIGURE 2:**
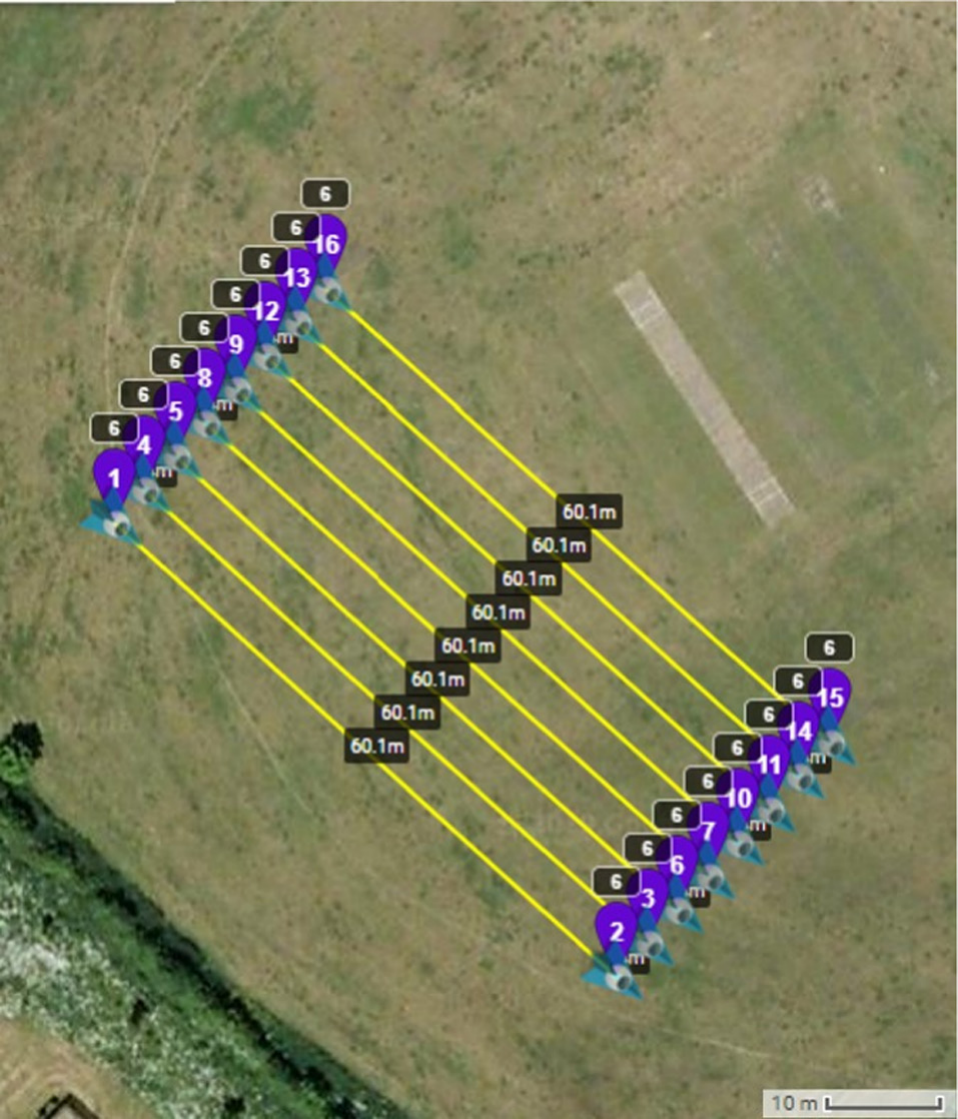
Drone's scanning pattern in a 30 m × 60 m area [52]

The drone's search pattern was based on autonomous flight operation by following a path of predetermined waypoints, as shown in Figure 2. DJI SPARK™ was able to detect the objects of interest by flying at a height of 6 m and at a speed of up to 7 kph, while it was decided that each scanning strip would overlap the adjacent strips of nearly 33% (i.e., 1 m) in order to offset any error regarding the positioning accuracy of the drone and thus ensure full coverage of the study area.

### 2.9 Implementation details

This section provides information about the sequence of the experiment steps and some parameters that were not included in the design of the experiment. In specific:

- Phases I and III were implemented in May (2019) during morning or afternoon hours. The weather was mostly sunny or partly cloudy with winds up to 18 kph, and therefore, the drone operation was not affected since DJI SPARK™ can withstand wind speeds of up to 28 kph [50]. It should be noted that the daylight created minimal shadowing effects as the objects were of “minimum” thickness (i.e., 2 mm, as mentioned in Section 2.3). Phase II was conducted in June (2019) under similar weather and daylight conditions.
- As each round (i.e., scenario) of the experiment was prepared by scattering the items in the area of interest, both the field team and the drone operator were in the adjacent parking site without having direct view to the sports pitch in order to avoid having prior knowledge of the objects' position.
- Both the field team and the drone operator were aware of the scanning patterns to be followed (as analyzed in Section 2.8) before entering the scene.
- Neither the field team nor the drone operator were aware of the number of the objects included in each scenario (as mentioned in Section 2.3).
- The time started to count when the field team or drone entered the scene and stopped when they left the scene. For this purpose, the northwest side of the rectangular‐shaped search area served as an entry/exit point.
- The time required to upload the CSV files containing the flight plan through the Litchi website (as mentioned in Section 2.6) is not counted since this process takes only 1–2 min and the preparation can be done prior to arrival on scene, as it really happened.
- When a searcher of the field team detected an object, he simply had to raise his hand and continue the search without having to stop to collect it.
- The success rate and the time required to fully search the area of interest were recorded for both the field team and the drone.
- As far as the field team, the number of objects identified per searcher was recorded, as well as the number of objects detected during the first scan of the area (i.e., before the searchers switched zones, as mentioned in Section 2.8).
- As far as the drone, the operator was asked to identify the color of the items, while the number of double‐detected objects due to overlapping was also recorded.
- The first round of experiments was conducted by the field team. Before these scenarios, the field team ran some trials in order to determine the size of objects that humans might have difficulty in detection, as explained in Section 2.3.
- It was found that the field team had difficulty in detection of black color after running the 12 multi‐colored scenarios of Phase I. Therefore, 4 additional scenarios containing black‐colored items were then carried out. The field team and the drone performed exactly the same scenarios.

## 3 RESULTS

### 3.1 Phase I

**FIGURE 3:**
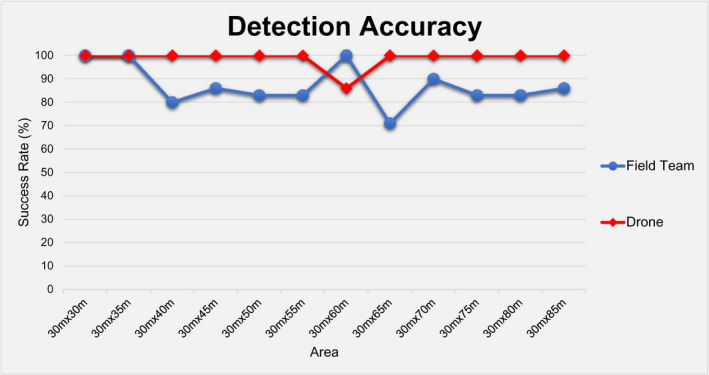
Detection accuracy of the field team and the drone

**FIGURE 4:**
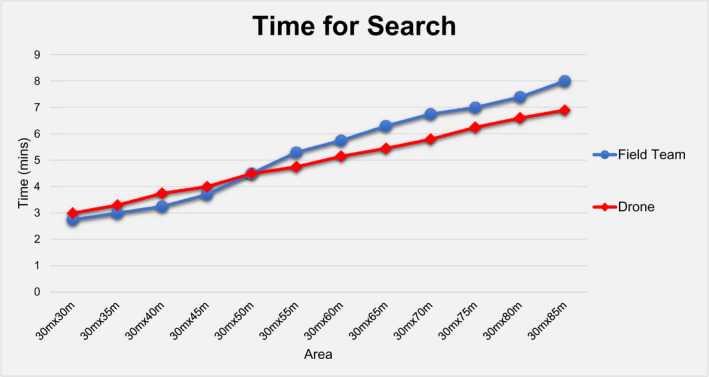
Time required for a full search of the area of interest for the field team and the drone

During this phase, the field team detected 70 out of total 80 objects by achieving 87.5% accuracy, while the drone missed only one detection by having a success rate of 99%, as shown in Table 1 and Figure 3. In addition, with regard to the speed of detection, the field team was faster by 10.7% in relatively small areas (up to 30 m × 45 m), while the drone was faster in areas larger than 30 m × 50 m by 12.1%, as shown in Table 1 and Figure 4.

**TABLE 1:** Detection accuracy and time required for a full search of the area of interest for the field team and the drone regarding the multi‐colored scenarios of phase I

		Success rate		Time for search (min)			
		Field team	Drone	Field team	Drone	Percentage difference	Average difference
Multi‐colored Scenarios	30m × 30m	100% (6/6)	100% (6/6)	2.75	3	9.1%	10.7%
	30m × 35m	100% (7/7)	100% (7/7)	3	3.3	10%	
	30m × 40m	80% (4/5)	100% (5/5)	3.25	3.75	15.4%	
	30m × 45m	86% (6/7)	100% (7/7)	3.7	4	8.1%	
	30m × 50m	83% (5/6)	100% (6/6)	4.5	4.5	0%	0%
	30m × 55m	83% (5/6)	100% (6/6)	5.3	4.75	−10.4%	−12.1%
	30m × 60m	100% (7/7)	86% (6/7)	5.75	5.15	−11.2%	
	30m × 65m	71% (5/7)	100% (7/7)	6.3	5.45	−13.5%	
	30m × 70m	90% (9/10)	100% (10/10)	6.75	5.8	−14.1%	
	30m × 75m	86% (6/7)	100% (7/7)	7	6.25	−10.7%	
	30m × 80m	80% (4/5)	100% (5/5)	7.4	6.6	−10.8%	
	30m × 85m	86% (6/7)	100% (7/7)	8	6.9	−13.8%	
	Total	87.5% (70/80)	99% (79/80)				

**FIGURE 5:**
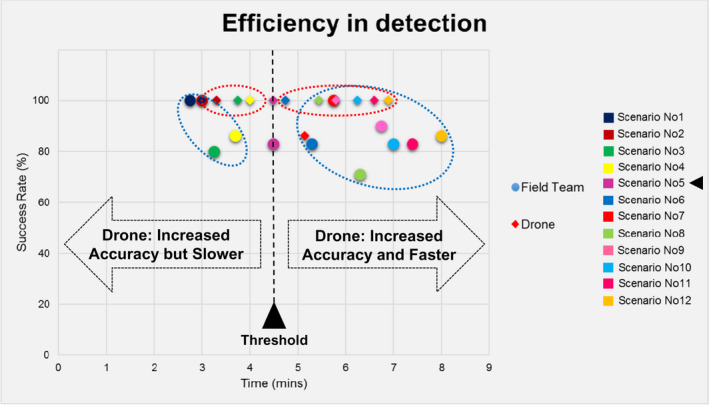
Combined view of the accuracy and time for search for the field team and the drone

The efficiency in detection is presented in Figure 5 as a combined view of the accuracy and time for detection. In specific, in this scatter chart, the success rate is plotted against the search time for each multi‐colored scenario of *Phase I* for both the field team and the drone operator.

The second part of *Phase I* consisted of 4 single‐colored scenarios, that is, objects of black color. By following a similar statistical approach to Part 1, Table 2 presents the accuracy and time for search for both the field team and the drone.

**TABLE 2:** Detection accuracy and time required for a full search of the area of interest for the field team and the drone regarding the single‐colored scenarios of phase I

		Success rate		Time for search (min)			
		Field team	Drone	Field team	Drone	Percentage difference	Average difference
Single‐colored Scenarios	30 m × 60 m	90% (9/10)	100% (10/10)	6	5.1	−15%	−15.5%
	30 m × 60 m	90% (9/10)	100% (10/10)	6.3	5.1	−19%	
	30 m × 85 m	80% (8/10)	100% (10/10)	8.2	7	−14.5%	
	30 m × 85 m	90% (9/10)	80% (8/10)	8.1	7	−13.5%	
	Total	87.5% (35/40)	95% (38/40)				

### 3.2 Phase II

The first area tested was covered with red clay soil, while the second area was covered with high grass in comparison with the short‐grass terrain of *Phase I*. Drone was able to reproduce the performance results obtained from the first phase, by achieving 100% in object detection, as shown in Table 3.

**TABLE 3:** Performance results of the drone in ERDA terrains

			Success rate	Time for search (min)
Scenarios	Red clay soil	30 m × 30 m	100% (6/6)	3
	
30 m × 60 m	100% (7/7)	5.15
	High grass	30 m × 30 m	100% (6/6)	3
		30 m × 60 m	100% (7/7)	5.15
		Total	100% (26/26)	

### 3.3 Phase III

The results obtained from the use of MATLAB computer vision toolbox for color‐based object detection are presented in Table 4. Unfortunately, this phase does not concern real‐time detection, as explained in Section 2.

**TABLE 4:** Efficiency of MATLAB in object detection based on color

		Success rate
Scenarios	Multi‐colored	100% (6/6)
	
100% (7/7)
	Single‐colored	100% (6/6)
		100% (7/7)
	Total	100% (120/120)

Moreover, it was found that MATLAB can detect objects (except black ones) that have a quarter of the original size (i.e., size: 2.5 cm × 2.5 cm) even when the DJI SPARK™ is flying four times faster than the project speed settings (i.e., speed: 28 kph), as shown in the figure below:

## 4 DISCUSSION

The use of UAVs in forensic applications for evidence detection tasks can be beneficial since these low‐cost and easy‐to‐use platforms can multidimensionally help crime scene or accident investigations and assist in apprehension and prosecution of offenders.

In specific, taking into account the results of *Phase I* (as presented in Section 3.1), it should be highlighted that drone deployment can achieve high detection rates of nearly 100%. However, it should be noted that the degree of drone's accuracy depends on the flight settings. Therefore, for the detection of such small objects (as described in Section 2.3), it was decided the drone would fly at a height of 6 m, at a speed of up to 7 kph, and with a window overlap of 33% (as mentioned in Section 2.8), where the changes result in an increase of the flight speed or altitude and/or a decrease of the degree of scan overlap could have a negative impact on achieving extremely high detection rates.

Additionally, it was found that drone could search relatively large areas faster by more than 10%. In specific, in areas larger than 30 m × 50 m, drone achieved a −12.1% and −15.5% in the time required for a full scan of the search area in the multi‐colored (Table 1, Figure 4) and single‐colored (Table 2) scenarios of *Phase I*, respectively. However, from a resource point of view, it should be highlighted that a 10% reduction in the time for search is equivalent to an approximately 50% reduction in the consumed man‐hours (Figure 7), as the field team consisted of 2 searchers compared to the sole drone operator.

By synthesizing the aforementioned findings, it can be deduced that the drone is considered to be ultimately more efficient for areas larger than 1,500 m^2^ (i.e., 30 m × 50 m area), since drone achieved superior performance results both in terms of accuracy and speed of detection compared to those obtained from the field team.

As far as the second phase, the reproducibility of the drone's performance results demonstrates the robustness of the proposed search method. In particular, the fact that drone achieved high detection rates in three different terrains (i.e., short grass, high grass, and red clay soil), as shown in Tables 1, 2, 3, provides reasonable support that drone deployment can ensure reliable detection capabilities. Furthermore, it is worth noting that the speed of detection for drone remained the same as the search width is not dependent on the terrain type. This proved to be significant when compared to the human approach, in which the swath size of the searcher is reduced. For example, the ground is covered by high grass in comparison with an asphalt terrain [53], as illustrated in Figure 8.

Moreover, a decrease in the degree of overlapping by increasing the track separation distance in the drone's scanning pattern will result in a reduction in the time for search but possibly at the expense of accuracy. Another way to achieve the same results is the utilization of cameras with larger field of view (e.g., fisheye lens cameras), but it must always be ensured that the potential positive results are not offset by the radial distortion [33, 54].

**FIGURE 6:**
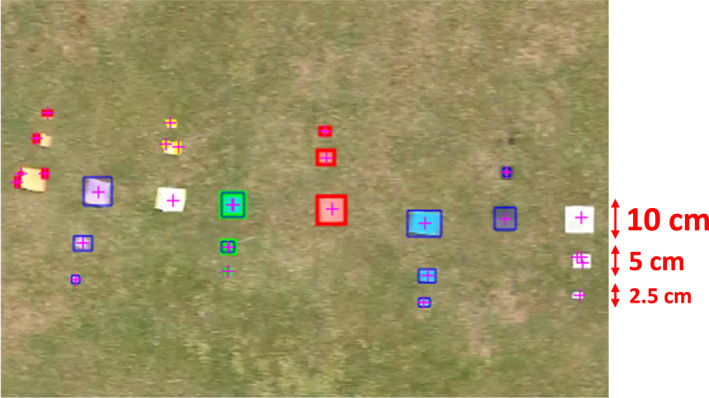
Color‐based detection capability of MATLAB when the drone is flying at 28 kph

Regarding *Phase III*, it should be highlighted that computer vision techniques can enhance the drone detection capabilities. Specifically, the fact that MATLAB ensured 100% accuracy in detection (Table 4), even when the drone flew faster and the objects were smaller (Figure 6), provides strong support that real‐time object detection based on computer vision techniques can be the key enabler of drone‐based forensic investigations, as is already the case, for example, in the field of autonomous driving systems [55, 56].

**FIGURE 7:**
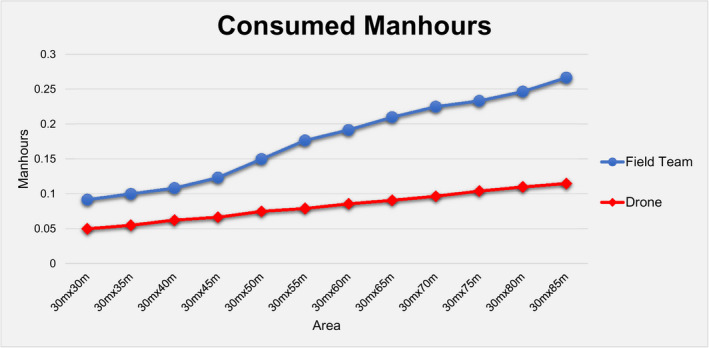
Consumed man‐hours for the field team and the drone

**FIGURE 8:**
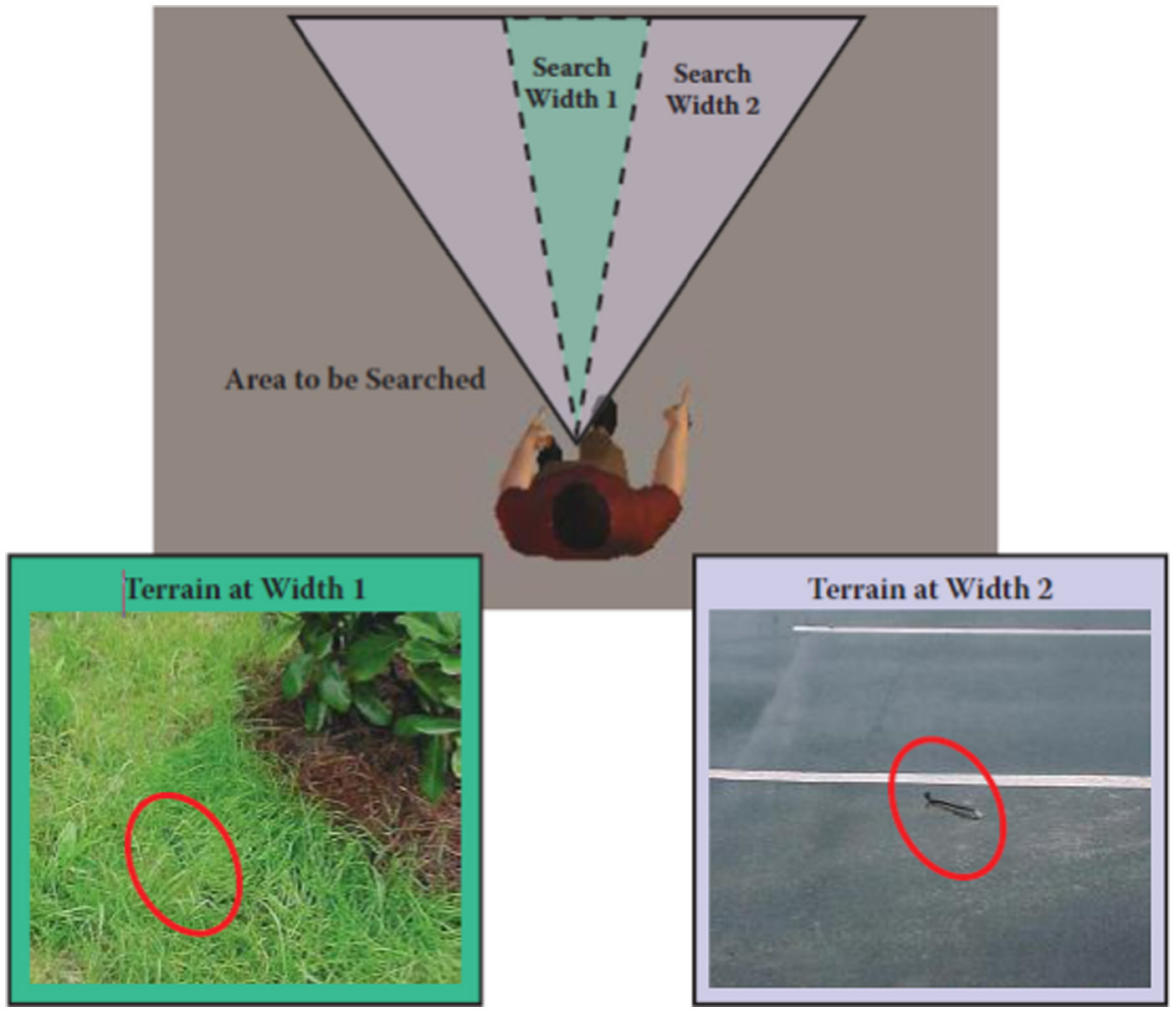
Search width varies depending on the terrain type [53]

However, it should be noted that color‐based detection by using computer vision techniques can result in increased rates of false alarms when, for example, a shadow is detected as a black object. The rate of false alarms depends on the ground complexity. In specific, a terrain of red clay soil with green vegetation, white stones, and shades of adjacent trees can lead to increased false alarms (i.e., red due to soil, green due to vegetation, white due to stones, black due to shadows) if the sensitivity settings (i.e., the color detection threshold) are not properly adjusted. Nevertheless, there are advanced software tools, such as the “TensorFlow Object Detection” [57], which can detect various types of objects such as vehicles, TV monitors, chairs, or even people. If these tools are enriched with forensic‐valuable objects (e.g., weapons and bomb components), they can be used to facilitate evidence detection without confronting the false alarms resulting from color‐based detection algorithms.

Apart from the above, it should be noted that drone deployment is adherent to communication data links since a connection loss or signal degradation results in a spatial discontinuity in the coverage of the area of interest. This was the case with the two missing detections in the single‐colored scenarios of *Phase I* (Table 2). Specifically, a blur distortion occurred as a result of the degradation of the RF signal between the drone and the SPARK™ remote controller, mostly due to the distance between them or secondarily due to interference from other nearby signals (Wi‐Fi IEEE 802.11 also operates in the same bandwidths with the controller, i.e., 2.4 GHz [49, 58]).

Lastly, it should be highlighted that the performance results for both the field team and the drone were obtained under conducive weather and daylight conditions, as discussed in Section 2.1. Moreover, the search area was free of spatial constraints such as trees, which could block the drone's accessibility capabilities or limit the visibility due to vegetation cover, while 4G signal and/or Wi‐Fi networks were available at all times, as mentioned in Section 2.2.

## 5 CONCLUSIONS AND FUTURE WORK

### 5.1 Conclusions

The current research project examined the usefulness of UAVs in real‐time evidence detection in outdoor crime scene investigations. Based on the obtained results, this project provides reasonable support that drone deployment as a forensic detection tool offers:

- Increased accuracy in detection compared to the traditional human approach. In specific, the drone can ensure detection rates of nearly 100%.
- Increased speed of detection in relatively large areas, since the drone requires less time to fully search these areas of interest compared to the traditional human approach.
- Reliable detection capabilities since the drone can achieve high detection rates over a range of terrain types.
- Enhanced detection capabilities through computer vision techniques. If interoperability between drones and computer vision techniques is achieved, the UAV‐based real‐time evidence detection will be more consistent with real‐life investigations.

### 5.2 Future work

The following ideas could be tested to further investigate the usefulness of UAVs as a forensic detection tool:

- The examination of drone's detection capabilities at night or in adverse weather conditions
- The determination of the drone's optimal flight settings (i.e., flight speed and altitude, degree of overlapping) for achieving high detection rates for objects smaller than those used in the current study
- The incorporation of computer vision techniques into UAV‐based real‐time detection. It should be noted that the combination of software tools for object recognition (e.g., “TensorFlow Object Detection” [57]) and the determination of the object's position in space, by calculating the GPS coordinates based on the detection time (in the case where the drone follows a non‐accelerated motion. For example, flying at a constant speed without reducing the speed at the predefined waypoints) or by using cameras with GPS‐tagging capabilities (e.g., “MAPIR” [59]), can lead to a “go‐to‐collect” approach in autonomous drone‐based forensic investigations.

## References

[1] PwC Belgium, Agoria vzw/asbl . Commercial applications. In: A drone’s eye view: overview of the Belgian UAV ecosystem and the development of commercial drone applications in Belgium. Diegem, Belgium: PwC Belgium; 2018. p. 20–37. https://www.pwc.be/en/documents/20180801‐drones‐eye‐view‐v2.pdf

[2] Horsman G . Unmanned aerial vehicles: a preliminary analysis of forensic challenges. Digit Investig. 2016;16:1–11. 10.1016/j.diin.2015.11.002

[3] Atkinson S , Carr G , Shaw C , Zargari S . Drone forensics: the impact and challenges. In: Montasari R , Jahankhani H , Hill R , Parkinson S , editors. Digital forensic investigation of internet of things (IoT) devices. Cham, Switzerland: Springer International Publishing; 2021. p. 65–124.

[4] Marshall DM , Barnhart RK , Shappee E , Most M . Chapter 1‐History. Chapter 2 ‐ UAS Applications. Chapter 3 ‐ The “System” in UAS. In: Introduction to unmanned aircraft systems. 2nd ed. Boca Raton, FL: CRC Press; 2016. p. 1–55. 10.1201/9781315372044

[5] Joint Air Power Competence Centre . Strategic concept of employment for unmanned aircraft systems in NATO. Kalkar, Germany: Joint Air Power Competence Centre; 2010. http://www.japcc.org/wp‐content/uploads/UAS_CONEMP.pdf

[6] Dörtbudak MF . Unmanned aerial vehicles (UAVs): a new tool in counterterrorism operations? In: Carapezza EM , editor. Proceedings SPIE volume 9456: sensors, and command, control, communications, and intelligence (C3I) Technologies for Homeland Security, defense, and law enforcement XIV; 2015 April 20‐22; Baltimore, MD. Bellingham, WA: SPIE – International Society for Optics and Photonics; 2015. 10.1117/12.2085373

[7] Carter D , Burris P , Brandt S . Fifth‐generation target drone phase I design. Proceedings of the 11th AIAA Aviation Technology, Integration, and Operations (ATIO) Conference; 2011 Sep 20‐22; Virginia Beach, VA. Reston, VA: American Institute for Aeronautics and Astronautics (AIAA); 2011. 10.2514/6.2011–7012

[8] Single European Sky ATM Research 3 Joint Undertaking . European drones outlook study: unlocking the value for Europe. Luxembourg: Publications Office of the EU; 2017. https://data.europa.eu/doi/10.2829/219851

[9] Tikanmäki I , Tuohimaa T , Rajamäki J . How and why unmanned aircraft vehicles can improve real‐time awareness? Int J Circuits Syst Signal Process. 2011;5:469–77.

[10] Shakhatreh H , Sawalmeh A , Al‐Fuqaha A , Dou Z , Almaita E , Khalil I , et al. Unmanned aerial vehicles: a survey on civil applications and key research challenges. IEEE Access. 2019;7:48572–634. 10.1109/ACCESS.2019.2909530

[11] Shahid Z , Rashid A . Applications of UAV in daily life. In: Proceedings of the 54th AIAA Aerospace Sciences Meeting; 2016 Jan 4‐8; San Diego, CA. Reston, VA: American Institute for Aeronautics and Astronautics (AIAA); 2016. 10.2514/6.2016-1895

[12] Kanistras K , Martins G , Rutherford MJ , Valavanis KP . A survey of unmanned aerial vehicles (UAVs) for traffic monitoring. Proceedings of the International Conference on Unmanned Aircraft Systems; 2013 May 28‐31; Atlanta, GA. Piscataway, NJ: IEEE; 2013. 10.1109/ICUAS.2013.6564694

[13] Puri A , Valavanis KP , Kontitsis M . Statistical profile generation for traffic monitoring using real‐time UAV based video data. In: Mediterranean Conference on Control and Automation; 2007 Jun 27–29; Athens, Greece. Piscataway, NJ: IEEE; 2007. 10.1109/MED.2007.4433658

[14] Stöcker C , Bennett R , Nex F , Gerke M , Schmidt C , Zein T . Deliverable 4.1 UAVs 4 LA: UAVs for land rights – A guide to regulatory practice. Enschede, Netherlands: Its4land Consortium; 2017. https://its4land.com/deliverables/

[15] Nex F , Remondino F . UAV for 3D mapping applications: a review. Appl Geomat. 2014;6(1):1–15. 10.1007/s12518-013-0120-x

[16] Pöllänen R , Toivonen H , Peräjärvi K , Karhunen T , Ilander T , Lehtinen J , et al. Radiation surveillance using an unmanned aerial vehicle. Appl Radiat Isot. 2009;67(2):340–4. 10.1016/j.apradiso.2008.10.008 19046635

[17] Briechle S , Sizov A , Tretyak O , Antropov V , Molitor N , Krzystek P . UAV‐based detection of unknown radioactive biomass deposits in Chernobyl’s exclusion zone. Int Arch Photogramm Remote Sens Spatial Inf Sci. 2018;42(2):163–9. 10.5194/isprs-archives-XLII-2-163-2018

[18] Faiçal B , Costa FG , Pessin G , Ueyama J , Freitas H , Colombo A , et al. The use of unmanned aerial vehicles and wireless sensor networks for spraying pesticides. J Syst Archit. 2014;60(4):393–404. 10.1016/j.sysarc.2014.01.004

[19] Yoo W , Yu E , Jung J . Drone delivery: factors affecting the public’s attitude and intention to adopt. Telemat Inform. 2018;35(6):1687–700. 10.1016/j.tele.2018.04.014

[20] Hwang J , Kim H , Kim W . Investigating motivated consumer innovativeness in the context of drone food delivery services. J Hosp Tour Manag. 2019;38:102–10. 10.1016/j.jhtm.2019.01.004

[21] Ackerman E , Koziol M . The blood is here: Zipline’s medical delivery drones are changing the game in Rwanda. IEEE Spectrum. 2019;56(5):24–31. 10.1109/MSPEC.2019.8701196

[22] Torabi A , Shafer MW , Vega GS , Rothfus KM . UAV‐RT: an SDR based aerial platform for wildlife tracking. Proceedings of the 2018 IEEE 88th Vehicular Technology Conference (VTC‐Fall); 2018 Aug 28‐30; Chicago, IL. Piscataway, NJ: IEEE; 2018. p. 1–6. 10.1109/VTCFall.2018.8690711

[23] Ward S , Hensler J , Alsalam B , Gonzalez LF . Autonomous UAVs wildlife detection using thermal imaging, predictive navigation and computer vision. Proceedings of the 2016 IEEE Aerospace Conference; 2016 May 5‐12; Big Sky, MT. Piscataway, NJ: IEEE; 2016. p. 1–8. 10.1109/AERO.2016.7500671

[24] Hodgson JC , Baylis SM , Mott R , Herrod A , Clarke R . Precision wildlife monitoring using unmanned aerial vehicles. Sci Rep. 2016;6:22574. 10.1038/srep22574 26986721 PMC4795075

[25] Fiori L , Doshi A , Martinez E , Orams MB , Bollard‐Breen B . The use of unmanned aerial systems in marine mammal research. Remote Sens. 2017;9(6):543. 10.3390/rs9060543

[26] Subhan B , Arafat D , Santoso P , Pahlevi K , Prabowo B , Taufik M , et al. Development of observing dolphin population method using small vertical take‐off and landing (VTOL) Unmanned Aerial System (AUV). 2019 IOP Conf Ser: Earth Environ Sci. 2019;278(1):12074. 10.1088/1755-1315/278/1/012074

[27] Grenzdörffer GJ , Engelb A , Teichertc B . The photogrammetric potential of low‐cost UAVs in forestry and agriculture. Int Arch Programm. 2008;37:1207–14.

[28] Torresan C , Berton A , Carotenuto F , Di Gennaro SF , Gioli B , Matese A , et al. Forestry applications of UAVs in Europe: a review. Int J Remote Sens. 2016;38(8–10):2427–47. 10.1080/01431161.2016.1252477

[29] Jonassen MO , Olafsson H , Agustsson H , Rognvaldsson O , Reuder J . Improving high‐resolution numerical weather simulations by assimilating data from an unmanned aerial system. Mon Weather Rev. 2012;140(11):3734–56. 10.1175/MWR-D-11-00344.1

[30] Cione JJ , Kalina EA , Uhlhorn EW , Farber AM , Damiano B . Coyote unmanned aircraft system observations in hurricane Edouard (2014). Earth Space Sci. 2016;3(9):370–80. 10.1002/2016EA000187

[31] Elston JS , Roadman J , Stachura M , Argrow B , Houston A , Frew E . The tempest unmanned aircraft system for in situ observations of tornadic supercells: Design and VORTEX2 flight results. J Field Robot. 2011;28(4):461–83. 10.1002/rob.20394

[32] Chand M. Effective usage of unmanned aerial vehicle technology in emergency operations in Canada [dissertation]. Victoria, British Columbia, Canada: Royal Roads University; 2018.

[33] Evers R , Masters P . The application of low‐altitude near‐infrared aerial photography for detecting clandestine burials using a UAV and low‐cost unmodified digital camera. Forensic Sci Int. 2018;289:408–18. 10.1016/j.forsciint.2018.06.020 30025566

[34] Isaacks MER The use of near‐infra remote sensing in the detection of clandestine human remains [dissertation]. San Marcos, TX: Texas State University; 2015.

[35] Mendis NDNA , Dharmarathne TSS , Wanasinghe NC . Use of unmanned aerial vehicles in crime scene investigations – Novel concept of crime scene investigations. Forensic Res Criminol Int J. 2017;4(1):1–2. 10.15406/frcij.2017.04.00094

[36] Mishra V , Dedhia H . Application of drones in the investigation and management of a crime scene. Glob J Res Anal. 2015;4(4):1–2.

[37] Pringle JK , Ruffell A , Jervis JR , Donnelly L , McKinley J , Hansen J , et al. The use of geoscience methods for terrestrial forensic searches. Earth Sci Rev. 2012;114(1–2):108–23. 10.1016/j.earscirev.2012.05.006

[38] Sharma BK , Chandra G , Mishra VP . Comparative analysis and implication of UAV and AI in forensic investigations. Proceedings of the 2019 Amity International Conference on Artificial Intelligence; 2019 Feb 4–6; Dubai, UAE. Piscataway, NJ: IEEE; 2019. p. 824–7. 10.1109/AICAI.2019.8701407

[39] Urbanová P , Jurda M , Vojtíšek T , Krajsa J . Using drone‐mounted cameras for on‐site body documentation: 3D mapping and active survey. Forensic Sci Int. 2017;281:52–62. 10.1016/j.forsciint.2017.10.027 29101908

[40] Butters O , Krosch M , Roberts M , Macgregor D . Application of forward‐looking infrared (FLIR) imaging from an unmanned aerial platform in the search for decomposing remains. J Forensic Sci. 2021;66(1):347–55. 10.1111/1556-4029.14581 32976643

[41] Rocke B , Ruffell A , Donnelly L . Drone aerial imagery for the simulation of a neonate burial based on the geoforensic search strategy (GSS). J Forensic Sci. 2021;66(4):1506–19. 10.1111/1556-4029.14690 33576508

[42] Gaszczak A , Breckon TP , Han J . Real‐time people and vehicle detection from UAV imagery. Proceedings SPIE Volume 7878: Intelligent Robots and Computer Vision XXVIII: Algorithms and Techniques; 2011 Jan 24‐25; San Francisco, CA. Bellingham, WA: SPIE – International Society for Optics and Photonics; 2011. 10.1117/12.876663

[43] Kamate S , Yilmazer N . Application of object detection and tracking techniques for unmanned aerial vehicles. Procedia Comput Sci. 2015;61:436–41. 10.1016/j.procs.2015.09.183

[44] Xu S , Savvaris A , He S , Shin H , Tsourdos A . Real‐time implementation of YOLO+JPDA for small scale UAV multiple object tracking. Proceedings of the 2018 International Conference on Unmanned Aircraft Systems; 2018 Jun 12–15; Dallas, TX. Piscataway, NJ: IEEE; 2018. p. 1336–41. 10.1109/ICUAS.2018.8453398

[45] Davenport GC . Remote sensing applications in forensic investigations. Hist Archeol. 2001;35(1):87–100. 10.1007/BF03374530

[46] Maio O , Endreny T , Lega M . Remote sensing for environmental forensics: thermal infrared images capture different surface temperatures in pollutant pools and dosed soils due to volatilization. Environ Forensics. 2017;18(2):101–9. 10.1080/15275922.2017.1304469

[47] Raymond NA , Card BL , Baker IL . A new forensics: developing standard remote sensing methodologies to detect and document mass atrocities. Genocide Studies Prevention. 2014;8(3):33–48. 10.5038/1911-9933.8.3.4

[48] Sharma RC , Kumar S , Kumar S , Mann M , Mayank SM . Photoacoustic remote sensing of suspicious objects for defence and forensic applications. Spectrochim Acta A Mol Biomol Spectrosc. 2020;224:117445. 10.1016/j.saa.2019.117445 31382229

[49] DJI . SPARK. [cited 2022 Jan 08]. Available form: https://www.dji.com/spark

[50] DJI . Fly Safe: GEO zone map. [cited 2022 Jan 08]. Available from: https://www.dji.com/gr/flysafe/geo‐map

[51] Google Maps . Defence Academy of the UK. [cited 2022 Jan 08]. Available from: https://goo.gl/maps/GvvvF8FA3MnzNUZR8

[52] Litchi . Mission hub. [cited 2022 Jan 08]. Available from: https://flylitchi.com/hub

[53] Gardner R , Krouskup D . Practical crime scene processing and investigation. 2nd ed. Hoboken, NJ: CRC Press; 2016.p. 108–12.

[54] Willneff J , Wenisch O . The calibration of wide‐angle lens cameras using perspective and non‐perspective projections in the context of real‐time tracking applications. Proceedings SPIE Volume 8085: Videometrics, Range Imaging, and Applications XI; 2011 Jun 21; Munich, Germany. Bellingham, WA: SPIE – International Society for Optics and Photonics; 2011. 10.1117/12.889331

[55] Dhall A , Dai D , Gool LV . Real‐time 3D traffic cone detection for autonomous driving. Proceedings of the IEEE Intelligent Vehicles Symposium (IV); 2019 Jun 9‐12; Paris, France. Piscataway, NJ: IEEE; 2019. p. 494–501. 10.1109/IVS.2019.8814089

[56] Pathak AR , Pandey M , Rautaray S . Application of deep learning for object detection. Procedia Comput Sci. 2018;132:1706–17. 10.1016/j.procs.2018.05.144

[57] DZone . Object detection tutorial in TensorFlow: real‐time object detection. [cited 2022 Jan 08]. Available from: https://dzone.com/articles/object‐detection‐tutorial‐in‐tensorflow‐real‐time

[58] Lee IG , Kim M . Interference‐aware self‐optimizing Wi‐fi for high efficiency internet of things in dense networks. Comput Commun. 2016;89‐90:60–74. 10.1016/j.comcom.2016.03.008

[59] MAPIR CAMERA . How‐to GPS tag images using GeoSetter. [cited 2022 Jan 08]. Available from: https://www.mapir.camera/blogs/guide/89765126‐how‐to‐gps‐tag‐images‐using‐geosetter

